# Identification of hub genes associated with prognosis, diagnosis, immune infiltration and therapeutic drug in liver cancer by integrated analysis

**DOI:** 10.1186/s40246-021-00341-4

**Published:** 2021-06-29

**Authors:** Xinyi Lei, Miao Zhang, Bingsheng Guan, Qiang Chen, Zhiyong Dong, Cunchuan Wang

**Affiliations:** 1 Department of Gastrointestinal Surgery, the First Affiliated Hospital of Jinan University, No.613 Huangpu Road West, Guangzhou, 510630 China; 2 Department of Respiratory, the First Affiliated Hospital of Jinan University, Guangzhou, 510630 China; 3 Department of Oncology, the First Affiliated Hospital of Jinan University, Guangzhou, 510630 China

**Keywords:** Liver cancer, Hub gene, Prognosis, Diagnosis, Immune infiltration, Therapeutic drug

## Abstract

**Background:**

Liver cancer is one of the most common cancers and causes of cancer death worldwide**.** The objective was to elucidate novel hub genes which were benefit for diagnosis, prognosis, and targeted therapy in liver cancer via integrated analysis.

**Methods:**

GSE84402, GSE101685, and GSE112791 were filtered from the Gene Expression Omnibus (GEO). Differentially expressed genes (DEGs) were identified by using the GEO2R. The GO and KEGG pathway of DEGs were analyzed in the DAVID. PPI and TF network of the DEGs were constructed by using the STRING, TRANSFAC, and Harmonizome. The relationship between hub genes and prognoses in liver cancer was analyzed in UALCAN based on The Cancer Genome Atlas (TCGA). The diagnostic value of hub genes was evaluated by ROC. The relationship between hub genes and tumor-infiltrate lymphocytes was analyzed in TIMER. The protein levels of hub genes were verified in HPA. The interaction between the hub genes and the drug were identified in DGIdb.

**Results:**

In total, 108 upregulated and 60 downregulated DEGs were enriched in 148 GO terms and 20 KEGG pathways. The mRNA levels and protein levels of *CDK1*, *HMMR*, *PTTG1*, and *TTK* were higher in liver cancer tissues compared to normal tissues, which showed excellent diagnostic and prognostic value. *CDK1*, *HMMR*, *PTTG1*, and *TTK* were positively correlated with tumor-infiltrate lymphocytes, which might involve tumor immune response. The *CDK1*, *HMMR,* and *TTK* had close interaction with anticancer agents.

**Conclusions:**

The *CDK1*, *HMMR*, *PTTG1*, and *TTK* were hub genes in liver cancer; hence, they might be potential biomarkers for diagnosis, prognosis, and targeted therapy of liver cancer.

## Highlights of our study

1. Three gene expression profiles (GSE84402, GSE101685, and GSE112791) were combined, for the first time, for integrated analysis in gene expression omnibus (GEO).
2. We revealed the interrelationship between the CDK1, HMMR, PTTG1, TTK, and immune infiltration.
3. CDK1, HMMR, PTTG1, and TTK could be identified as the novel biomarkers for prognosis and diagnosis in liver cancer.
4. We demonstrated the interaction between the CDK1, HMMR, TTK, and new types of anticancer agents and traditional chemotherapy drugs.

## Introduction

In the most common malignant tumor, liver cancer is one of the most common cancers and causes of cancer death worldwide, especially in China [1]. Liver cancer includes two histological types of malignant tumors: hepatocellular carcinoma (HCC) and intrahepatic cholangiocarcinoma (ICC) [2]. More than 840,000 new cases of liver cancer occurred in addition to 781,000 deaths in 2018, which had become a severe public health issue [3]. Liver cancer is mainly caused by the hepatitis B virus (HBV) and the hepatitis C virus (HCV) [4]. Meanwhile, aflatoxin, algal hepatoxins, betel nut, alcohol, and tobacco have been reported as potential risk factors of liver cancer [5, 6].

A comprehensive understanding of the occurrence, development, and metastasis of liver cancer will be beneficial for early diagnosis and precise treatment of patients. The immune checkpoint inhibitor (ICI) therapy targeting cytotoxic T-lymphocyte-associated protein-4 (CTLA-4), anti-programmed cell death protein-1 (PD-1), and programmed cell death-ligand 1 (PD-L1) were potential activity against HCC and manageable safety in clinical trial [7]. The molecular ablation of 3-phosphoinositide-dependent protein kinase-1 function can improve the susceptibility of HCC cells to be resistant to radiotherapy, which is related to deactivated PI3K/AKT/mTOR signaling way [8]. Recent meta-analysis has revealed that circulating tumor DNA (ctDNA) can serve as an assistant tool when combined with alpha-fetoprotein (AFP) for HCC detection [9]. The latest sequence studies have revealed that the special non-coding RNA, such as lncRNA NEAT1, lncRNA FLJ33360, lncRNA FOXD3-AS1, and lncRNA LEF1-AS1 are associated with liver cancer [10–13].

With the deepening understanding of epidemiology, etiology, and molecular biology of liver cancer, the regimens currently available were still unsatisfactory. Early diagnosis and precise treatment of liver cancer is still a huge challenge. Microarray technology has been widely used to detect the expression of genes in animals and humans, and it can also be helpful in exploring the change of gene expression during tumor occurrence and development. However, it is very difficult to acquire convincing results with the only one gene microarray analysis. In our study, three gene expression profiles (GSE84402, GSE101685, and GSE112791) were combined, for the first time, for integrated analysis in Gene Expression Omnibus (GEO). The differentially expressed genes (DEGs) were identified in liver cancer tissues compared to normal liver tissues. A large number of biomarkers have been identified in liver cancer; however, most of the biomarkers are directly experimental and not prospectively evaluated. In our research, Gene Ontology (GO) and Kyoto Encyclopedia of Genes and Genomes (KEGG) pathway analysis of DEGs were analyzed in the Database for Annotation, Visualization, and Integrated Discovery (DAVID). The protein-protein interaction (PPI) network was built by using the STRING database and cytoscape software to extract the hub genes and significant module. The transcription factors (TF) network was constructed by using the TRANSFAC, Harmonizome database, and cytoscape software. The prognostic roles of hub genes were verified in The Cancer Genome Atlas (TCGA) by using the UALCAN. The diagnostic value of hub genes in distinguishing between liver cancer tissues and normal liver tissues were analyzed by using the receiver operating characteristic (ROC) curve. The correlations between the hub genes and tumor-infiltrate lymphocytes were analyzed in the Tumor IMmune Estimation Resource (TIMER). The protein levels of hub genes were verified in the Human Protein Atlas (HPA). The interactions between hub genes and related therapeutic drugs were explored through the drug-gene interaction database (DGIdb). The hub genes might be targeted therapeutically or prioritized for drug progress. Due to a single database and few samples, the inconsistent results might appear. All our results were obtained from the multi-database which included sufficient samples to overcome the disadvantages. Our objective is to provide further understanding of the etiopathogenesis of liver cancer and identify the novel diagnostic indicators, prognostic markers, and precise target drug points by integrated analysis.

## Material and methods

### Data extraction

In total, three gene expression profiles (GSE84402, GSE101685, and GSE112791) were filtered from the Gene Expression Omnibus (GEO https:// www.ncbi.nlm.nih.gov/geo). As a free public genome, GEO database was utilized for storing array data and sequence data. The GSE84402 contained 14 liver cancer tissues and 14 matched corresponding non-cancerous liver tissues [14]. The GSE101685 included 24 liver cancer tissues and 8 normal liver tissues. The GSE112791 covered 15 normal liver tissues and 183 liver cancer tissues [15].

### Data processing

The differentially expressed genes (DEGs) between liver cancer tissues and normal liver tissues of GSE84402, GSE101685, and GSE112791 were screened out by using GEO2R (https://www.ncbi.nlm.nih.gov/geo/geo2r), respectively. The GEO2R is an interactive online tool based on the R programming language and is used for screening DEGs from the gene expression profiles between liver cancer tissues and normal liver tissues. The adjusted *P* value (adjust P) < 0.05 and |log_2_ fold change| > 2 were used to identify DEGs. The DEGs that were consistently expressed in three datasets were screened out.

### Analysis of functional and pathway enrichment

The Database for Annotation, Visualization and Integrated Discovery (DAVID version 6.8 https://david.ncifcrf.gov/tools.jsp) was used for analyzing the Gene Ontology (GO) and the Kyoto Gene and Genome Encyclopedia (KEGG) pathway of DEGs [16, 17]. *P*<0.05 was set as the cut-off criterion.

### Analysis of PPI network and TF network

The STRING (version 11.0 https://string-db.org) was utilized to analyze the functional interaction of DEGs [18]. The score of confidence > 0.7 was considered as significant value. The cytoscape (version 3.7.1) was used to build Protein-Protein interaction (PPI) network. The parameter settings were as follows: degree cutoff =2, node score cutoff = 0.2, k-score = 2, maximum depth = 100 [19]. The degree of genes > 10 were considered as hub genes. The significant module was screened by Molecular Complex Detection (MCODE). The curated transcription factor (TF) targets of DEGs were obtained from the TRANSFAC (http://gene-regulation.com/pub/databases.html) [20, 21] and Harmonizome database (https://maayanlab.cloud/Harmonizome/) [22]. The cytoscape software (version 3.7.1) was used to build TF network. The Fisher’s exact test was used to perform the enrichment analyses of DEGs. *P*<0.05 was set as the cut-off criterion. The *P* values were adjusted for multiple testing by the Bonferroni method.

### Verification and survival analysis of hub genes

The expression levels and survival analysis of hub genes were analyzed by using the UALCAN (http://ualcan.path.uab.edu/) which is a tool for analysis data from The Cancer Genome Atlas (TCGA) [23]. Based on transcripts per million (TPM) of hub genes, the data of liver cancer patients was divided into two groups. The high group’s TPM was higher than the upper quartile. The low/medium group’s TPM was lower than the upper quartile. The Kaplan-Meier and log-rank test were utilized for survival analysis. *P*<0.05 was set as the cut-off criterion. UALCAN was used to screen hub genes with potential prognostic value for subsequent analysis.

### Verification of hub genes by ROC analysis

The expression levels of hub genes with potential prognostic value were used for receiver operating characteristic (ROC) analysis to evaluate their diagnostic value to distinguish between liver cancer tissues and normal liver tissues in internal set (GSE84402) and an independent external set (GSE14520). The GSE14520 covered 21 normal liver tissues and 22 liver cancer tissues [24–30]. ROC analysis was performed in RStudio by pROC package [31]. The hub genes with area under curve (AUC) > 0.8 as well as *P* < 0.05 were set as the cut-off criterion.

### Immune infiltrates analysis of hub genes

Tumor IMmune Estimation Resource (TIMER, https://cistrome.shinyapps.io/timer/) was used to analyze the immune infiltrates across different types of cancer [32]. TIMER can analyze the abundance of immune cells from the gene expression in cancer samples. By applying the deconvolution method, TIMER was used to analyze the relationship between the infiltrating level of immune cells and the potential prognostic hub gene in liver cancer. The correlation between the potential prognostic hub genes and the gene markers for immune cell infiltration was performed through related modules. The relationship between somatic copy number alterations (SCNA) of the potential prognostic hub genes and infiltrating immune cells were explored via related modules. *P* < 0.05 was set as the cut-off criterion.

### Immunohistochemical analysis of hub genes in HPA

The protein levels of the potential prognostic hub gene in liver cancer tissues and normal liver tissues were extracted from the Human Protein Atlas (HPA, https://www.ptroteinatlas.org/) which contained the data of immunohistochemistry expression for human tissues [33]. The levels of expression were divided into four groups: high, medium, low, and not detected via the score system, which included the proportion of stained cells (> 75%, 25–75%, or < 25%) and the intensity of staining (strong, moderate, weak, or negative).

### Drug-gene interaction analysis of hub genes

The potential prognostic hub genes were supposed as the promising drug targets for searching drugs through the Drug**-**Gene Interaction database (DGIdb, version 4. 0.2**-**sha1 afd9f30b, https://dgidb.genome.wustl.edu/) [34]. The DGIdb consists of the drug**-**gene interaction data from the Drug Bank, ChEMBL, NCBI Entrez, Ensembl, PharmGKB, PubChem, clinical trial, and literature in PubMed, which can help researchers mine existing data and generate assumptions about how genes may be targeted therapeutically or prioritized for drug development [35]. The cytoscape (version 3.7.1) was applied to perform the drug-gene interaction network.

## Results

### Identification of DEGs in liver cancer

**Fig. 1:**
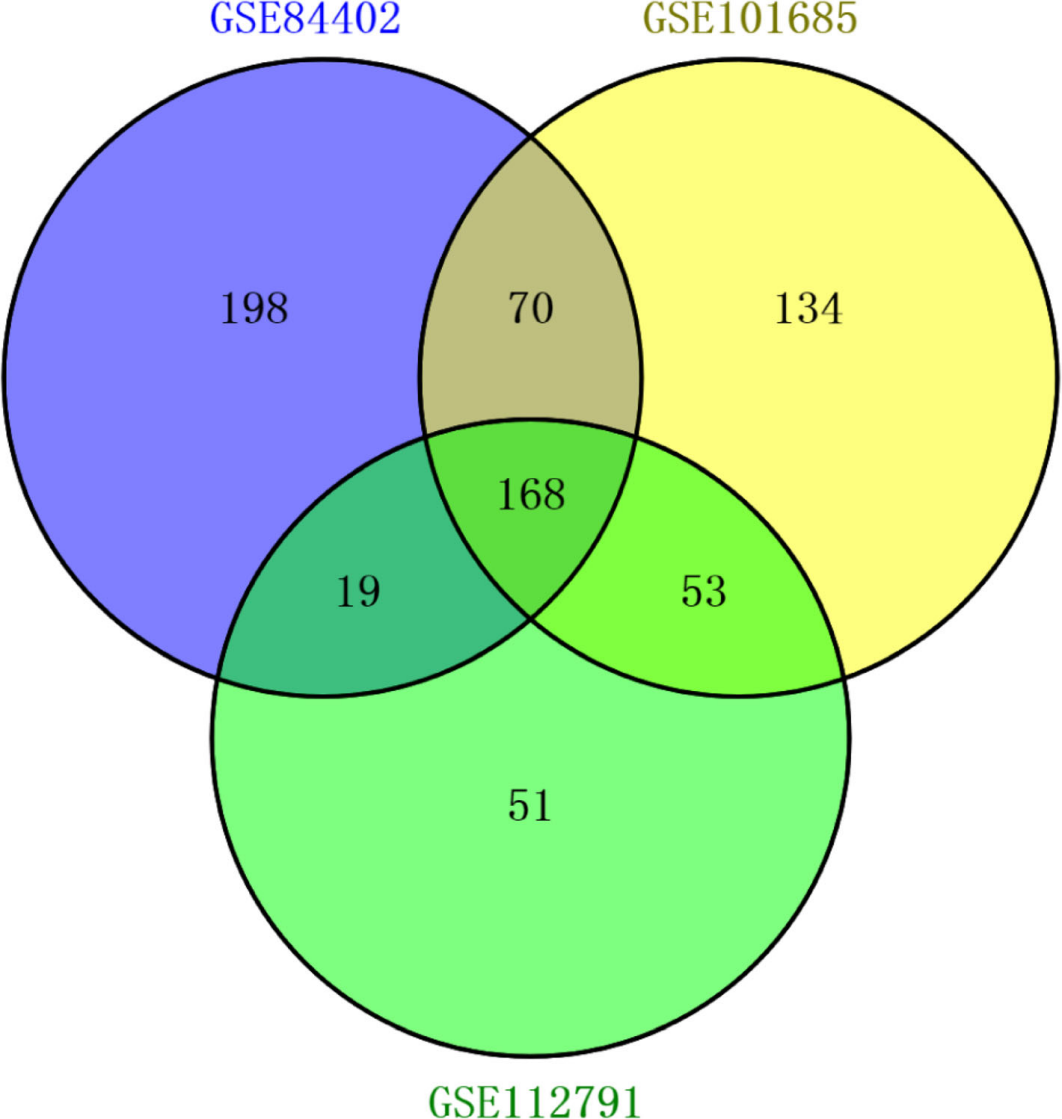
Identification of differentially expression genes (DEGs) in three mRNA expression profiles

In total, 455, 425, and 291 DEGs were extracted from the GSE84402, GSE101685, and GSE112791 datasets, respectively. In total, 168 DEGs were consistently expressed in the three datasets (Fig. 1), and they included 60 upregulated DEGs and 108 downregulated DEGs (Table 1).

**Table 1:** DEGs in liver cancer samples compared with normal samples

DEGs	Gene name
Upregulated	***CCNB1***, ***CDKN3***, ***CCNB2***, ***ASPM***, ***TOP2A***, ***UBE2T***, ***BIRC5***, ***FAM83D***, *MDK*, ***KIF4A***, ***CDK1***, *FAM72A*//*/FAM72D*///*FAM72B*///*FAM72C*, ***TTK***, ***ANLN***, ***CENPF***, ***NCAPG***, ***PBK***, ***PTTG1***, ***AURKA***, ***RACGAP1***, *GPC3*, ***NUF2***, ***PRC1***, *GINS1*, ***GMNN***, ***RRM2***, ***MELK***, ***NEK2***, ***CDC20***, *IQGAP3*, ***NDC80***, ***DLGAP5***, ***ECT2***, ***HMMR***, ***KIF20A***, *SULT1C2*, ***RAD51AP1***, *IGF2BP3*, ***CENPK***, ***DTL***, *DUXAP10*, ***HELLS***, *FLVCR1*, ***TYMS***, *CAP2*, ***MAD2L1***, ***UHRF1***, ***KIAA0101***, ***MCM6***, *PRR11*, ***BUB1B***, *ACSL4*, *PEG10*, *CRNDE*, *ROBO1*, *SLC35F6*///*CENPA*, *CD24*, *CTHRC1*, ***CKAP2***, *RBM24*.
Downregulated	*CLEC4M*, *CLEC4G*, *OIT3*, *FCN3*, *CYP2A6*, *TTC36*, *FOSB*, *CYP1A2*, *APOF*, *FOS*, *FCN2*, *MT1G*, *GLS2*, *GBA3*, *LCAT*, *CLEC1B*, *KCNN2*, *CXCL14*, *GLYAT*, *HGF*, *MARCO*, *CYP39A1*, *MT1X*, *GPM6A*, *HHIP*, *ANXA10*, *C8A*, *MT1M*, *TMEM27*, *GYS2*, *AKR1D1*, *HAMP*, *MT1F*, *PLAC8*, *CYP2B6*, *C7*, *KMO*, *MT1H*, *NAT2*, *SLC22A1*, *CYP2A7*, *CNDP1*, *MT1HL1*, *C3P1*, *BCO2*, *CRHBP*, *MT1E*, *GSTZ1*, *KCND3*, *SORL1*, *HAO2*, *ADH1B*, *CYP4A11*, *EGR1*, *ESR1*, *SLC25A47*, *CYP4A22*///*CYP4A11*, *GNMT*, *HGFAC*, *LINC01093*, *F9*, *SRPX*, *LINC00844*, *HEPN1*///*HEPACAM*, *SRD5A2*, *CYP2B7P*///*CYP2B6*, *MFSD2A*, *FLJ22763*, *FOLH1*, *BBOX1*, *SDS*, *BCHE*, *C9*, *SLC10A1*, *TSLP*, *LYVE1*, *MME*, *PGLYRP2*, *DCN*, *CYP3A4*, *CYP8B1*, *KBTBD11*, *GHR*, *CTH*, *CFHR4*, *AADAT*, *CXCL2*, *CYP26A1*, *C6*, *CETP*, *PDGFRA*, *FBP1*, *SERPINE1*, *RSPO3*, *PBLD*, *RDH16*, *SLCO1B3*, *IDO2*, *PZP*, *LPA*, *PCK1*, *AFM*, *ASPA*, *CLRN3*, *CNTN3*, *HPGD*, *ACSM3*, *LOC101928916*///*NNMT*.

A total of 60 upregulated DEGs and 108 downregulated DEGs were identified in the liver cancer tissues, compared with normal liver tissues. The hub genes were shown in boldface

### GO analysis and KEGG pathway of DEGs in liver cancer

**Fig. 2:**
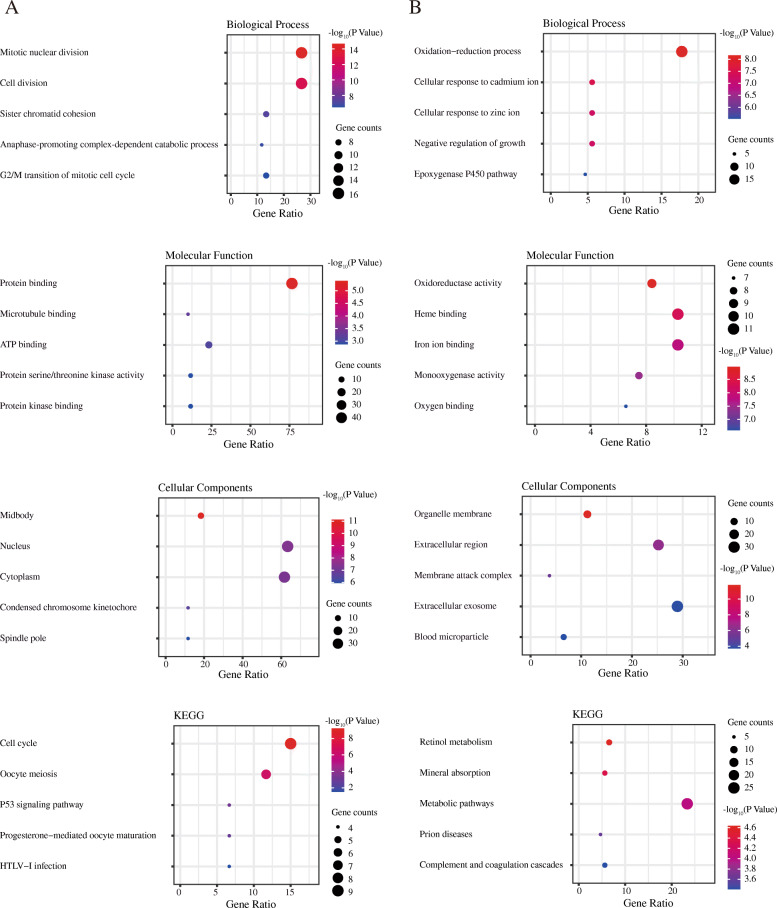
Gene Ontology (GO) and Kyoto Encyclopedia of Genes and Genomes (KEGG) pathway of DEGs. **a** GO and KEGG pathway analyses of upregulated genes. **b** GO and KEGG pathway analyses of downregulated genes. The GO terms and KEGG pathways were ranked by −log_10_(*P* value). Top 5 terms were selected according to −log_10_(*P* value). Gene counts: the number of enriched genes in each term. Gene ratio: the ratio of the number of enriched genes in each term to the total number of DEGs. BP: Biological Process. MF: Molecular Function. CC: Cellular Components

The GO and KEGG pathway of DEGs was performed by using the DAVID 6.8. The DEGs were divided into biological process groups, molecular function groups, cellular components groups, and KEGG pathway groups. The GO terms and KEGG pathways were ranked by −log_10_(*P* value). Top 5 GO terms and KEGG pathways were selected according to −log_10_(*P* value). Figure 2 shows the top 5 GO terms and KEGG pathways for upregulated DEGs (Fig. 2a) and downregulated DEGs (Fig. 2b).

### PPI network and significant module analysis in liver cancer

**Fig. 3:**
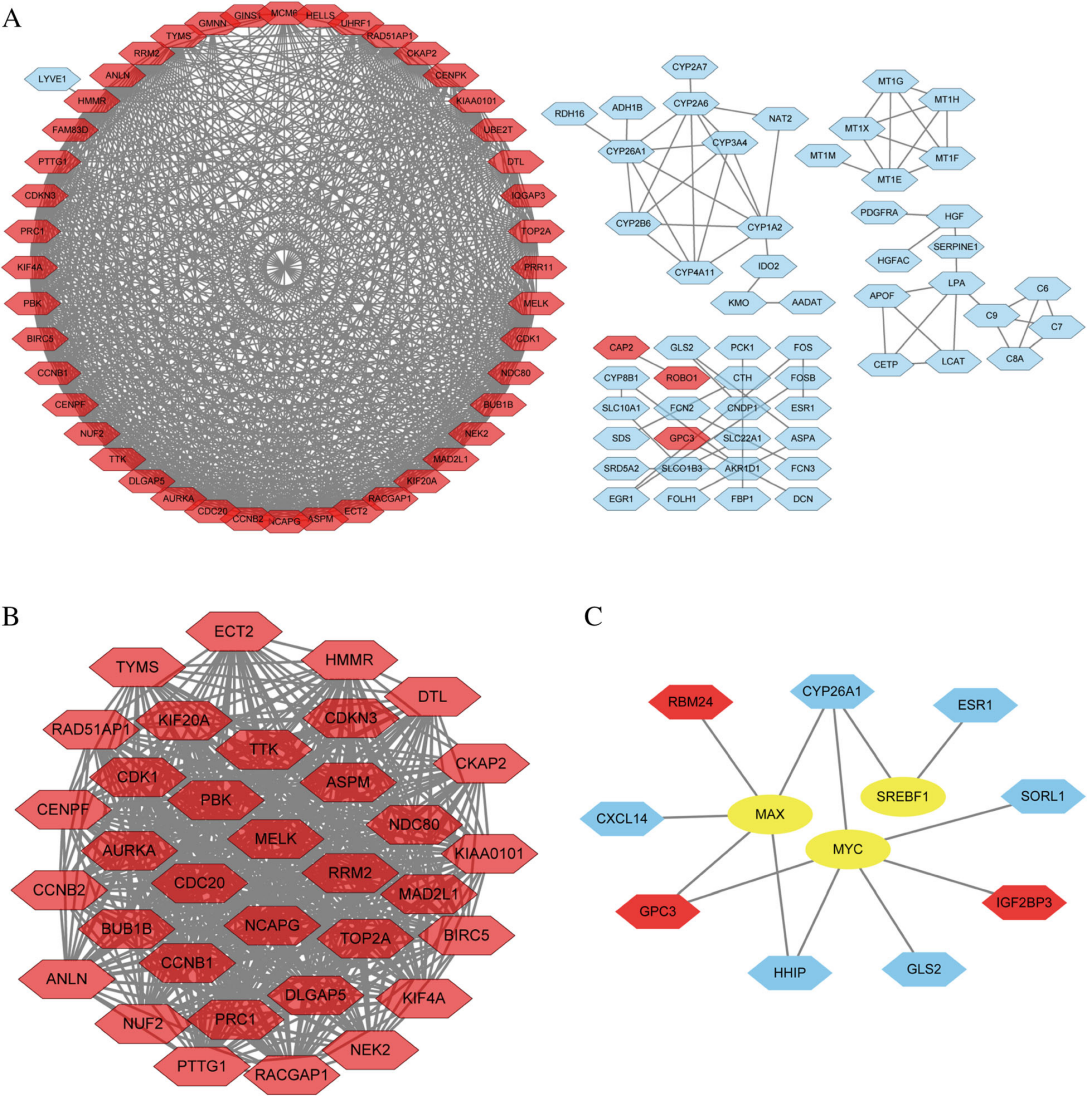
Protein-protein interaction (PPI) network and transcription factor (TF) network of DEGs. **a** PPI network contained 100 nodes and 738 sides. **b** Significant module was selected from PPI network. **c** TF network contained 12 nodes and 13 sides. Red nodes represented upregulated genes. Blue nodes represented downregulated genes. Yellow node represented transcription factors. The line represented interaction relationship between nodes

In total, 100 genes (score of confidence > 0.7) in 168 DEGs were filtered into the PPI network. The PPI network included 100 nodes and 738 sides. It consisted of 47 upregulated genes and 53 downregulated genes (Fig. 3a). In total, 41 genes (degree > 10) were considered as hub genes (Table 1, in bold). The characteristics of hub genes are shown in Table 2, which consisted of degree, betweenness centrality, closeness centrality, clustering coefficient, stress, and average shortest path length. The significant module was chosen from the PPI network by analysis of MCODE (Fig. 3b). The TF network comprised 9 DEGs and 3 TFs (Fig. 3c).

**Table 2:** The topology properties of 41 hub genes (The genes are ranked by degree)

Genes	Degree	Betweenness centrality	Closeness centrality	Clustering coefficient	Stress	Average shortest path length
*CDC20*	41	4.09E-02	9.36E-01	7.48E-01	446	1.07
*CDK1*	40	2.89E-02	9.17E-01	7.79E-01	398	1.09
*TOP2A*	40	3.73E-02	9.17E-01	7.67E-01	394	1.09
*BUB1B*	39	1.65E-02	8.98E-01	7.99E-01	320	1.11
*CCNB2*	39	1.06E-02	8.98E-01	8.19E-01	284	1.11
*NCAPG*	39	2.64E-02	8.98E-01	7.96E-01	354	1.11
*ASPM*	39	1.50E-02	8.98E-01	8.08E-01	302	1.11
*AURKA*	38	8.26E-03	8.80E-01	8.45E-01	232	1.14
*MAD2L1*	38	7.61E-03	8.80E-01	8.48E-01	224	1.14
*CCNB1*	38	7.61E-03	8.80E-01	8.48E-01	224	1.14
*DLGAP5*	37	9.50E-02	8.63E-01	8.45E-01	216	1.16
*KIF20A*	37	1.05E-02	8.63E-01	8.54E-01	211	1.16
*PBK*	37	5.79E-03	8.63E-01	8.74E-01	180	1.16
*CENPF*	37	1.16E-02	8.63E-01	8.44E-01	222	1.16
*RRM2*	37	5.51E-03	8.63E-01	8.75E-01	174	1.16
*MELK*	37	6.29E-03	8.63E-01	8.72E-01	178	1.16
*NDC80*	36	4.01E-03	8.46E-01	8.98E-01	134	1.18
*PTTG1*	35	3.72E-03	8.30E-01	9.08E-01	116	1.20
*BIRC5*	35	3.14E-03	8.30E-01	9.18E-01	102	1.20
*TTK*	35	2.84E-03	8.30E-01	9.23E-01	96	1.20
*PRC1*	35	2.20E-03	8.30E-01	9.29E-01	88	1.20
*HMMR*	35	4.80E-02	8.30E-01	9.81E-01	350	1.20
*KIAA0101*	35	5.88E-03	8.30E-01	8.81E-01	148	1.20
*DTL*	35	6.38E-03	8.30E-01	8.74E-01	158	1.20
*NEK2*	34	3.29E-03	8.15E-01	9.20E-01	96	1.23
*NUF2*	34	2.92E-03	8.15E-01	9.20E-01	94	1.23
*CDKN3*	34	3.07E-03	8.15E-01	9.23E-01	90	1.23
*RACGAP1*	33	9.43E-04	8.00E-01	9.62E-01	42	1.25
*KIF4A*	33	2.22E-03	8.00E-01	9.45E-01	62	1.25
*RAD51AP1*	32	7.50E-04	7.86E-01	9.68E-01	34	1.27
*ECT2*	31	2.28E-04	7.72E-01	9.87E-01	12	1.30
*ANLN*	31	2.20E-04	7.72E-01	9.87E-01	12	1.30
*TYMS*	30	1.19E-03	7.59E-01	9.52E-01	46	1.32
*CKAP2*	27	0.00	7.21E-01	1.00	0	1.39
*MCM6*	25	1.14E-02	6.88E-01	8.30E-01	130	1.45
*UHRF1*	19	1.07E-03	6.29E-01	9.36E-01	22	1.60
*UBE2T*	19	0.00	6.29E-01	1.00	0	1.60
*CENPK*	14	0.00	5.87E-01	1.00	0	1.70
*GMNN*	13	9.00E-05	5.79E-01	9.74E-01	4	1.73
*FAM83D*	12	0.00	5.71E-01	1.00	0	1.75
*HELLS*	11	2.63E-04	5.64E-01	9.09E-01	10	1.77

### Verification and survival analysis of hub genes in liver cancer

**Fig. 4:**
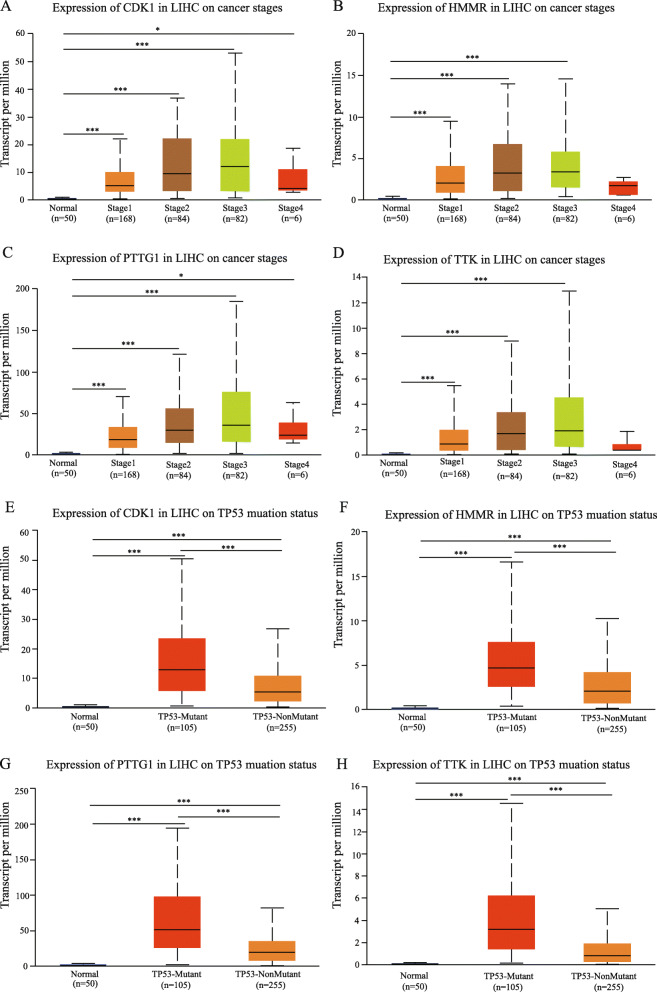
The mRNA expression levels of prognostic hub gene in liver hepatocellular carcinoma (LIHC) in subgroup analyses. The published online data of gene mRNA expression level were analyzed by UALCAN platform. Subgroup analyses were performed based on patients’ LIHC stages (**a**–**d**) and TP53 mutation status (**e–h**). T test was performed on the relevant results (*P<0.05, **P<0.01,***P<0.001)

The UALCAN online was used for analyzing the expression of significant hub genes in TCGA between liver hepatocellular carcinoma samples and normal liver samples. The trend of expression of significant hub genes was similar to the results generated by GEO datasets (Fig. 4). Meanwhile, the association between the mRNA expression of significant hub genes and clinical characteristics of liver hepatocellular carcinoma patients was analyzed via using the UALCAN, including the patient’s cancer stages and TP53 mutation status. The mRNA expression of *CDK1*, *HMMR*, *PTTG1*, and *TTK* were associated with advanced stages of liver hepatocellular carcinoma. Liver hepatocellular carcinoma patients who were with advanced cancer stages inclined to have the higher mRNA expression levels of *CDK1*, *HMMR*, *PTTG1*, and *TTK* (Fig. 4a–d). The expression levels of *CDK1*, *HMMR*, *PTTG1*, and *TTK* in stage 3 was higher than those in stage 4, which were attributed to the limited number of stage 4 patients (only six patients in stage 4). The higher mRNA expression levels of *CDK1* (*P* < 0.0001), *HMMR* (*P* < 0.0001), *PTTG1* (*P* < 0.0001), and *TTK* (*P* < 0.0001) were explored in liver hepatocellular carcinoma patients with TP53 mutation (Fig. 4e–h).

**Fig. 5:**
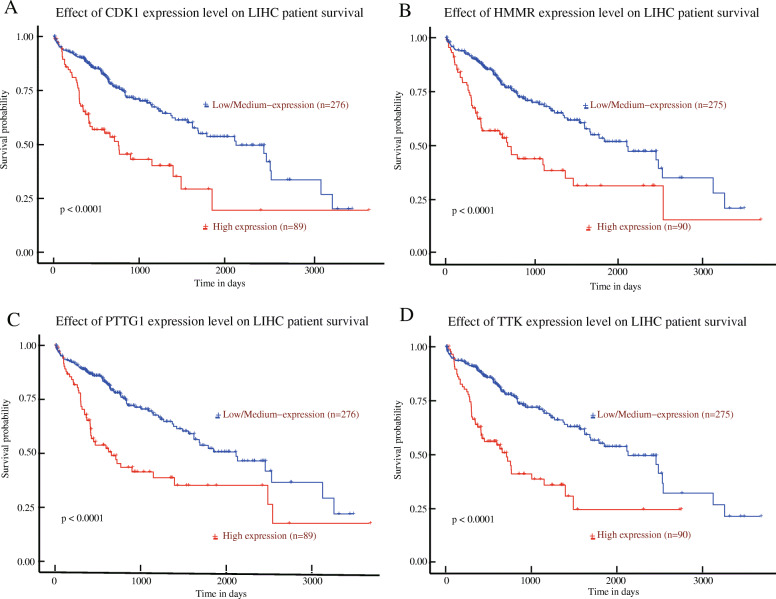
Overall survival analysis of prognostic hub genes in liver hepatocellular carcinoma (LIHC) was performed by using the UALCAN platform. Survival analysis curve for CDK1 (**a**), HMMR (**b**), PTTG1 (**c**), and TTK (**d**) in patients with LIHC from The Cancer Genome Atlas (TCGA). Log-rank test was performed on the relevant results

The survival analysis of liver hepatocellular carcinoma patients in TCGA were performed based on hub genes by using the UALCAN. The results revealed that high expression of *CDK1* (*P* < 0.0001), *HMMR* (*P* < 0.0001), *PTTG1* (*P* < 0.0001), and *TTK* (*P* < 0.0001) were associated with shorter overall survival rates (Fig. 5a–d). In summary, *CDK1*, *HMMR*, *PTTG1*, and *TTK* might be potential biomarkers to evaluate the prognosis of liver hepatocellular carcinoma patients.

### Verification of hub genes by ROC analysis

**Fig. 6:**
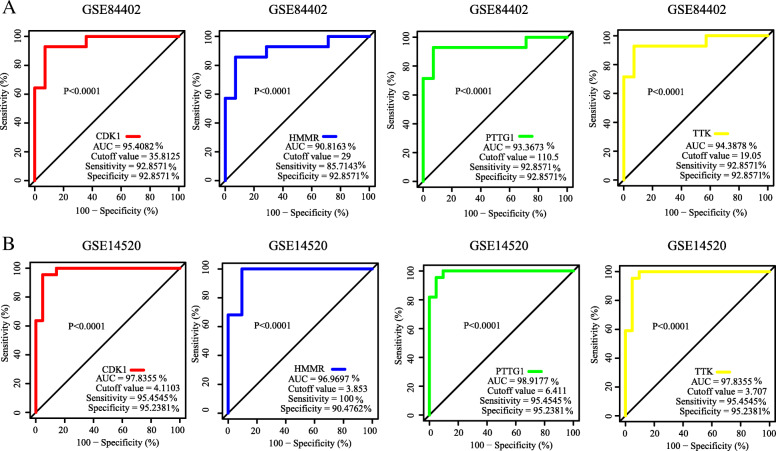
Receiver operating characteristic (ROC) curves analysis was implemented to evaluate the diagnostic value of four hub genes to distinguish between liver cancer tissues and liver normal tissues. **a** ROC curves to evaluate the diagnostic efficiency of the CDK1, HMMR, PTTG1, and TTK in internal set (GSE84402) to distinguish between liver cancer tissues and liver normal tissues. **b** ROC curves to evaluate the diagnostic efficiency of the CDK1, HMMR, PTTG1, and TTK in external set (GSE14520) to distinguish between liver cancer tissues and liver normal tissues

To identify the diagnostic value of *CDK1*, *HMMR*, *PTTG1*, and *TTK* distinguishing between liver cancer tissues and normal liver tissues, ROC analysis was performed by utilizing the data of the internal set (GSE84402). As shown in Fig. 6a, the AUC was 0.95 (*P* < 0.0001) for *CDK1*, 0.91 (*P* < 0.0001) for *HMMR*, 0.93 (*P* < 0.0001) for *PTTG1*, and 0.94 (*P* < 0.0001) for *TTK*. In the independent external set (GSE14520), the AUC was 0.98 (*P* < 0.0001) for *CDK1*, 0.97 (*P* < 0.0001) for *HMMR*, 0.99 (*P* < 0.0001) for *PTTG1*, and 0.98 (*P* < 0.0001) for *TTK* (Fig. 6b). Thus, the four hub genes might be potential diagnostic biomarker of liver cancer.

### Immune infiltrates analysis of hub genes

**Fig. 7:**
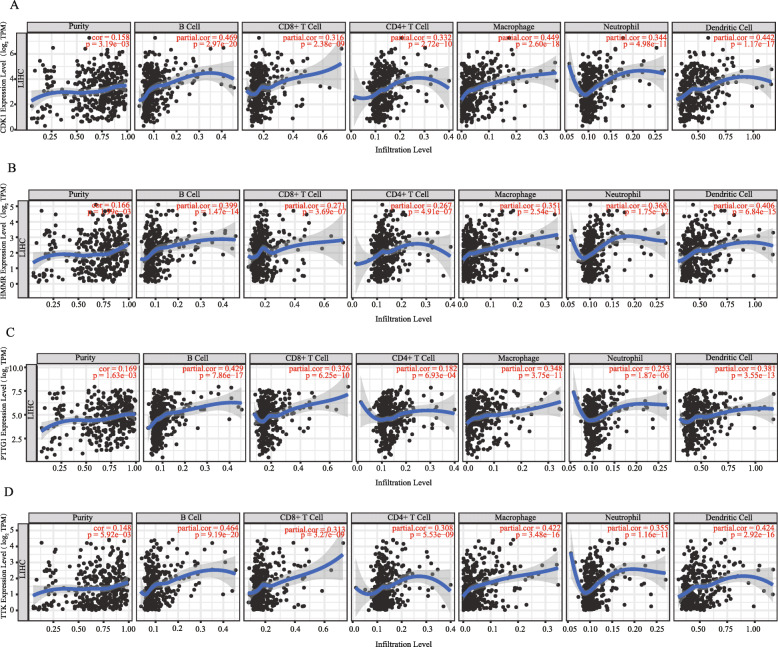
Correlation between four hub gene expression and infiltration levels of immune cells in liver hepatocellular carcinoma (LIHC). **a** CDK1 expression was significantly positively correlated with tumor purity and infiltrating levels of B cells, CD8+ T cells, CD4+ T cells, macrophages, neutrophils, and dendritic cells in LIHC. **b** HMMR expression was significantly positively correlated with tumor purity and infiltrating levels of B cells, CD8+ T cells, CD4+ T cells, macrophages, neutrophils, and dendritic cells in LIHC. **c** PTTG1 expression was significantly positively correlated with tumor purity and infiltrating levels of B cells, CD8+ T cells, CD4+ T cells, macrophages, neutrophils, and dendritic cells in LIHC. **d** TTK expression was significantly positively correlated with tumor purity and infiltrating levels of B cells, CD8+ T cells, CD4+ T cells, macrophages, neutrophils, and dendritic cells in LIHC

The correlation between the mRNA expression of *CDK1*, *HMMR*, *PTTG1*, *TTK*, and infiltrating immune cells in liver cancer was analyzed by using the TIMER database. *CDK1* showed significant correlation with the abundance of B cell (cor = 0.469, *P* = 2.97e−20), CD8+ T cell (cor = 0.316, *P* = 2.38e−9), CD4+ T cell (cor = 0.332, *P* = 2.72e−10), macrophage (cor = 0.449, *P* = 2.60e−18), neutrophil (cor = 0.344, *P* = 4.98e−11), and dendritic cell (cor = 0.442, *P* = 1.17e−17) (Fig. 7a). *HMMR* showed significant correlation with the abundance of B cell (cor = 0.399, *P* = 1.47e−14), CD8+ T cell (cor = 0.271, *P* = 3.69e−7), CD4+ T cell (cor = 0.267, *P* = 4.91e−7), macrophage (cor = 0.351, *P* = 2.54e−11), neutrophil (cor = 0.368, *P* = 1.75e−12), and dendritic cell (cor = 0.406, *P* = 6.84e−15) (Fig. 7b). *PTTG1* showed significant correlation with the abundance of B cell (cor = 0.429, *P* = 7.86e−17 ), CD8+ T cell (cor = 0.326, *P* = 6.25e−10), CD4+ T cell (cor = 0.182, *P* = 6.93e−4), macrophage (cor = 0.348, *P* = 3.75e−11), neutrophil (cor = 0.253, *P* = 1.87e−6), and dendritic cell (cor = 0.381, *P* = 3.55e−13) (Fig. 7c). *TTK* showed significant correlation with the abundance of B cell (cor = 0.464, *P* = 9.19e−20), CD8+ T cell (cor = 0.313, *P* = 3.27e−9), CD4+ T cell (cor = 0.308, *P* = 5.53e−9), macrophage (cor =0.422, *P* = 3.48e−16), neutrophil (cor = 0.355, *P* = 1.16e−11), and dendritic cell (cor = 0.424, *P* = 2.92e−16) (Fig. 7d). These results provided strong evidence that *CDK1*, *HMMR*, *PTTG1*, and *TTK* played crucial roles for infiltrating immune cells, including B cells, CD8+ T cells, CD4+ T cells, macrophages, neutrophils, and dendritic cells.

**Fig. 8:**
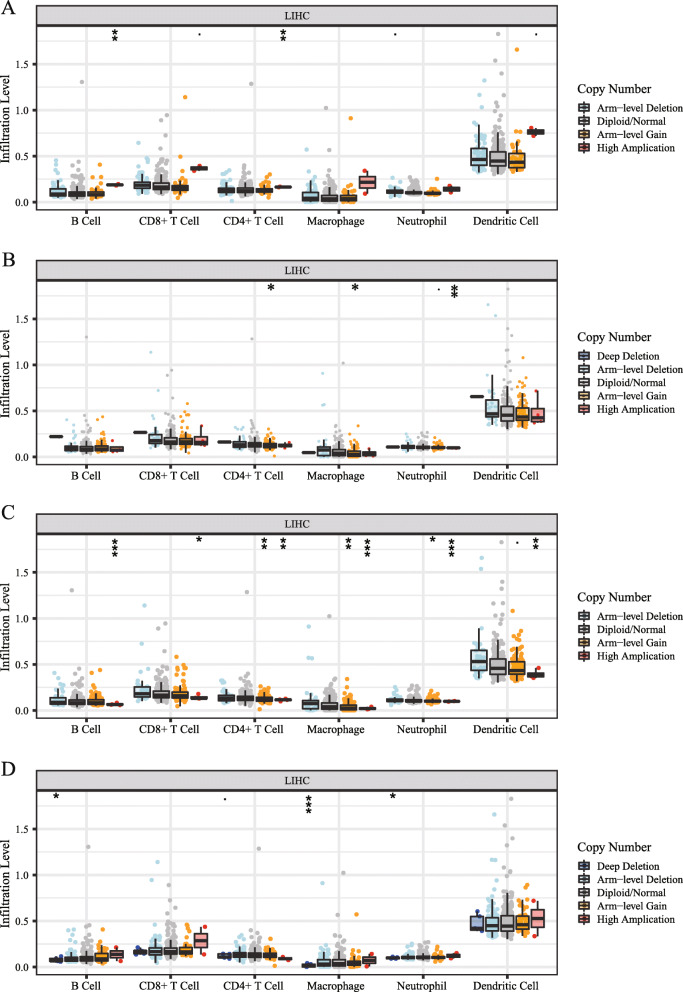
The association of somatic copy number alterations (SCNA) of four hub genes with immune cell infiltration in liver hepatocellular carcinoma (LIHC). **a** SCNA of CDK1with infiltrating levels of B cells, CD8+ T cells, CD4+ T cells, macrophages, neutrophils, and dendritic cells in LIHC. **b** SCNA of HMMR with infiltrating levels of B cells, CD8+ T cells, CD4+ T cells, macrophages, neutrophils, and dendritic cells in LIHC. **c** SCNA of PTTG1 with infiltrating levels of B cells, CD8+ T cells, CD4+ T cells, macrophages, neutrophils, and dendritic cells in LIHC. **d** SCNA of TTK with infiltrating levels of B cells, CD8+ T cells, CD4+ T cells, macrophages, neutrophils, and dendritic cells in LIHC. SCNA of hub genes were divided into five levels, including deep deletion, arm-level deletion, normal, arm-level gain, and high amplification

The somatic copy number alterations (SCNA) included deep deletion, arm-level deletion, diploid/normal, arm-level gain, and high amplification. Furthermore, the relationship between SCNA of the *CDK1, HMMR, PTTG1*, and *TTK* and infiltrating immune cells in liver cancer was explored via using TIMER. These results showed that the copy number alterations (CNA) of *CDK1* had significant correlation with the infiltrating levels of B cells and CD4+ T cells (Fig. 8a); the CNA of *HMMR* had significant correlation with the infiltrating levels of CD4+ T cells, macrophages, and neutrophils (Fig. 8b); the CNA of *PTTG1* had significant correlation with the infiltrating levels of B cells, CD8+ T cells, CD4+ T cells, macrophages, neutrophils and dendritic cells (Fig. 8c); the CNA of *TTK* had significant correlation with the infiltrating levels of B cells, macrophages, and neutrophils (Fig. 8d).

The correlation between *CDK1*, *HMMR*, *PTTG1*, and *TTK* and gene markers for different subsets of immune cells in liver cancer were analyzed through the TIMER-related modules. As shown in Table 3, the expression levels of *CDK1*, *HMMR*, *PTTG1*, and *TTK* were significantly associated with most of the immune markers of immune cells, except for natural killer cells.

**Table 3:** Correlation analysis between *CDK1*, *HMMR*, *PTTG1*, and *TTK* and related immune markers in immune cells, as evaluated using TIMER

Description	Gene marker	***CDK1***		***HMMR***		***PTTG1***		***TTK***	
		Cor	P	Cor	P	Cor	P	Cor	P
CD8+ T cell	CD8A	0.198	***	0.139	**	0.164	**	0.188	***
	CD8B	0.184	***	0.096	0.064	0.226	***	0.165	**
T cell (general)	CD3D	0.274	***	0.154	**	0.333	***	0.266	***
	CD3E	0.202	***	0.120	*	0.175	***	0.195	***
	CD2	0.216	***	0.121	*	0.204	***	0.204	***
B cell	CD19	0.273	***	0.204	***	0.247	***	0.273	***
	CD79A	0.158	**	0.065	0.212	0.116	*	0.177	***
Monocyte	CD86	0.284	***	0.266	***	0.250	***	0.283	***
	CD115	0.131	*	0.138	**	0.102	*	0.119	*
TAM	CCL2	0.039	0.459	0.023	0.656	− 0.023	0.665	0.021	0.682
	CD68	0.230	***	0.186	***	0.149	*	0.204	***
	IL10	0.219	***	0.223	***	0.156	*	0.235	***
M1 macrophage	NOS2	− 0.020	0.695	0.080	0.123	− 0.140	**	− 0.005	0.926
	IRF5	0.394	***	0.407	***	0.291	***	0.373	***
	COX2	0.101	0.053	0.071	0.171	− 0.025	0.637	0.094	0.070
M2 Macrophage	CD163	0.067	0.197	0.153	**	− 0.022	0.676	0.073	0.160
	VSIG4	0.080	0.123	0.138	**	0.024	0.647	0.059	0.255
	MS4A4A	0.089	0.086	0.142	**	0.027	0.601	0.079	0.128
Neutrophils	CD66b	0.123	*	0.096	0.066	0.094	0.071	0.107	*
	CD11b	0.257	***	0.333	***	0.237	***	0.280	***
	CCR7	0.089	0.086	0.045	0.385	− 0.030	0.569	0.070	0.181
Natural killer cell	KIR3DL1	0.010	0.843	0.068	0.189	− 0.053	0.308	0.009	0.869
	KIR2DL1	− 0.035	0.500	− 0.023	0.656	− 0.068	0.190	− 0.044	0.393
	KIR2DS4	0.075	0.148	0.076	0.142	0.029	0.581	0.044	0.400
Dendritic cell	CD11C	0.331	***	0.310	***	0.246	***	0.326	***
	CD1C	0.121	*	0.066	0.204	0.041	0.434	0.104	*
	NRP1	0.231	***	0.195	***	0.005	0.916	0.180	***
PDL1	CD274	0.209	***	0.334	***	0.062	0.230	0.062	0.230
Th l	STAT4	0.262	***	0.179	***	0.215	***	0.246	***
	STAT1	0.372	***	0.372	***	0.248	***	0.398	***
	TBX21	0.081	0.119	0.050	0.341	0.018	0.736	0.084	0.107
	CD4	0.222	***	0.232	***	0.123	*	0.228	***
	IFNG	0.266	***	0.219	***	0.285	***	0.275	***
Th 2	GATA3	0.203	***	0.135	**	0.133	*	0.185	***
	STAT6	0.114	*	0.192	***	− 0.108	*	0.115	*
	CXCR4	0.315	***	0.224	***	0.204	***	0.292	***
	CCR4	0.206	***	0.220	***	− 0.023	0.663	0.213	***
Treg	FOXP3	0.163	**	0.271	***	0.023	0.655	0.205	***
	CCR8	0.392	***	0.418	***	0.209	***	0.403	***
	STAT5B	0.247	***	0.355	***	− 0.022	0.676	0.278	***
	TGFB1	0.278	***	0.174	***	0.207	***	0.253	***
T cell exhaustion	PD-1	0.330	***	0.196	***	0.333	***	0.308	***
	CTLA4	0.357	***	0.244	***	0.384	***	0.348	***
	LAG3	0.294	***	0.205	***	0.348	***	0.318	***
	TIM-3	0.296	***	0.281	***	0.272	***	0.289	***
	GZMB	0.092	0.078	0.048	0.355	0.123	*	0.065	0.212

*P < 0.05; **P < 0.01; ***P < 0.0001

### Immunohistochemical analysis of hub genes in HPA

**Fig. 9:**
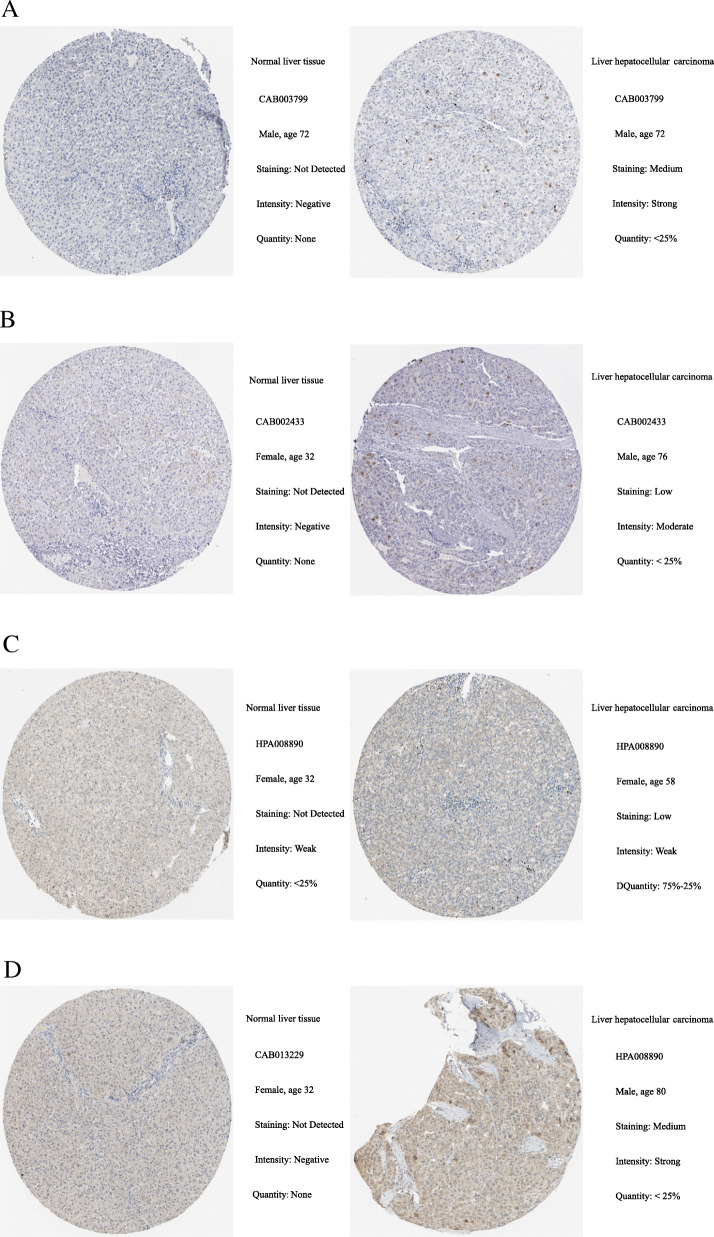
Immunohistochemistry (IHC) of four hub genes based on the Human Protein Atlas (HPA). **a** Protein levels of CDK1 in normal liver tissue and liver hepatocellular carcinoma tissue. **b** Protein levels of HMMR in normal liver tissue and liver hepatocellular carcinoma tissue. **c** Protein levels of PTTG1 in normal liver tissue and liver hepatocellular carcinoma tissue. **d** Protein levels of TTK in normal liver tissue and liver hepatocellular carcinoma tissue

Based on the protein expression data from the HPA, the protein expression levels of *CDK1*, *HMMR*, *PTTG1*, and *TTK* in liver cancer tissues and normal liver tissues were compared by utilizing the antibodiesCAB003799, CAB002433, HPA008890, and CAB013229. The immunohistochemistry results confirmed that the protein expression levels of *CDK1*, *HMMR*, *PTTG1*, and *TTK* were higher in liver cancer tissues than normal liver tissues (Fig. 9).

### Drug-gene interaction analysis of hub genes in DGIdb

**Fig. 10:**
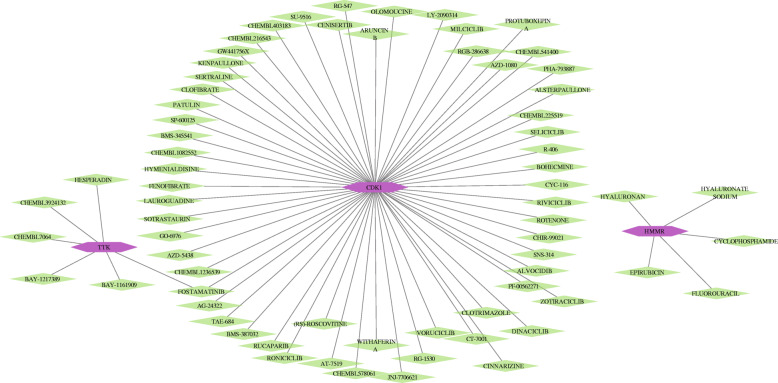
Drug-hub gene interaction network. The drug-hub gene interaction network contained 72 nodes and 70 sides. Purple nodes represented hub genes. Green nodes represented the drug. The line represented interaction relationship between the hub genes and the drug

DGIdb was utilized to analyze the drugs that potentially interacted with the four hub genes (*CDK1*, *HMMR*, *PTTG1*, and *TTK*). Through the DGIdb, 69 drugs interacted with *CDK1*, *HMMR*, and *TTK*, which might help develop new treatment target for liver cancer therapy (Fig. 10).

## Discussion

However, the past 30 years had been characterized by a broadening of understanding of liver cancer’s pathogenesis and an advance in diagnostic and therapeutic strategies for managing liver cancer patients, the clinical outcome remained poor [36]. Liver cancer had become a serious global health issue due to the current regimens having limited efficacy in liver cancer patients. Meanwhile, searching for specific molecular biomarkers for development and metastasis of liver cancer had important significance in the diagnosis and therapy of liver cancer patients.

In this study, 60 upregulated genes and 108 downregulated genes were identified by bioinformatics method in three expression profiles. The GO and KEGG pathway analysis of DEGs in liver cancer revealed that DEGs were enriched in the function and pathway related to the occurrence and development of liver cancer. Downregulated genes were enriched in the pathway of retinol metabolism, which was associated with the development of liver cancer. The decrease of retinol storage in the liver was observed in hepatocellular carcinoma patients [37]. A recent study revealed that the apoptosis pathway (Bax/Caspase) and cell cycle arrest pathway (P53/P21) could be activated after exposure to the alternating low-intensity and intermediate-frequency electric field in hepatocellular carcinoma spheroids [38]. The study of hepatocellular carcinoma cell lines was similar to our study in that it had shown that hepatocellular carcinoma cells displayed a downregulated metabolic pathway and complement coagulation cascades [39]. Meanwhile, in our study, the role of P450 pathway in the progression of liver cancer was crucial which was similar to the previous studies [40–42]. Interestingly, human T-lymphotropic virus type I (HTLV-1) infection had been identified to be the significant pathway in our study. Previous studies pointed out that HTLV-1 may be associated with the development of the hepatitis C virus infection [43]. As was known to all, the hepatitis C virus is linked to the development of liver cancer. Emerging studies had found prion disease to be pathological aggregation in malignant tumors related to misfolded p53, a tumor-suppressor protein. The prion-like behavior of oncogenic P53 mutants appeared to be a direct correlation to tumorigenesis [44]. Interestingly, in our study, the downregulated genes were enriched in the pathway of prion disease. The role of prion disease pathway is worthy of further investigation.

In this study, 3 significant TFs were screened from the TF regulatory network, including MAX, MYC, and SREBF1, which played important roles in the formation and development of tumors [45, 46]. In total, 41 hub genes were extracted out from the DEGs by analyzing the PPI network. The mRNA expression of 4 hub genes, including*CDK1*, *HMMR*, *PTTG1*, and *TTK*, were significantly associated with the survival probability of liver cancer patients in TCGA. Moreover, the above 4 hub genes were validated by performing ROC analysis in the external set GSE14520. As a result, these four hub genes showed the excellent diagnostic value for liver cancer, which were consistent with the results of the internal set GSE84402. These results suggested that *CDK1*, *HMMR*, *PTTG1*, and *TTK* could be the diagnostic biomarkers in liver cancer to distinguish between cancer tissues and normal tissues.

The cyclin-dependent kinase 1 (*CDK1*), known as cell division control protein 2, is required for the transition from the G2 phase into mitosis [47]. The *CDK1*-cyclinB complex allowed *CDK1* to phosphorylate more than one hundred proteins, which promoted nuclear envelope breakdown, chromatin condensation, and spindle assembly [48]. Recent researches had revealed that the expression of *CDK1* was high in different types of carcinomas, such as thyroid cancer, pancreatic ductal adenocarcinoma, colorectal cancer, and ovarian cancer and so on [49–52]. The mouse knockout experiments had indicated that *CDK1* was essential for mammalian cell proliferation; only *CDK1* could initiate the onset of mitosis [53]. Prior research revealed that *CDK1* activity was dysregulated by direct genetic alteration in tumorigenesis. Meanwhile, the derangement of P53 pathway or of DNA damage checkpoints indirectly could result in the deregulation of *CDK1* [54, 55]. As the previous study identified, *CDK1* was overexpressed in hepatocellular carcinoma and was related to the development of tumor through the CDK1/PDK1/β-Catenin pathway, which could predict worse survival outcomes [56, 57].

In our study, the mRNA expression levels and protein levels of *CDK1* were higher in liver cancer samples than normal liver samples; meanwhile, the mRNA expression levels of *CDK1* were associated with advanced cancer stages and TP53 mutation. Liver hepatocellular carcinoma patients with high expression levels of *CDK1* were associated with lower overall survival rates. These results indicated that *CDK1* was a prognostic biomarker in liver cancer. *CDK1* SCNA was closely relevant to immune cell infiltration level, and further analysis revealed that *CDK1* expression was positively correlated with the infiltration levels of B cells, CD8+ T cells, CD4+ T cells, macrophages, neutrophils, and dendritic cells. The correlation between *CDK1* expression and immune cell gene markers revealed that *CDK1* regulates liver cancer tumor immunity through multiple immune cell populations. Our results suggested that high expression levels of *CDK1* could increase immune activation and cytotoxicity of the immune system in liver cancer by increasing the infiltration of immune cells. We inferred that *CDK1* might be involved in the occurrence and development of liver cancer by regulating the P53 pathway and immune system. Due to the lack of evidence on the immunologic mechanism of *CDK1*, the immunologic mechanism of *CDK1* is worthy of further testing.

The hyaluronan-mediated motility receptor (*HMMR*) is identified as a hyaluronan receptor purified from the supernatants of murine cells [58]. The prior study had shown that the *HMMR* was crucial for the spindle to align correctly; even the few mice without *HMMR* were able to survive or many suffered from deformed and underdeveloped brains [59–61]. In our study, the biological process results had shown that the *HMMR* was enriched in transition of mitotic cell cycle. Extensive research had identified that the *HMMR* was overexpressed in non-small cell lung cancer, stomach cancer, bladder cancer, etc. [62–64]. The expression levels of the *HMMR* might be a specific prognostic marker in terms of progressions-free survival in papillary muscle-invasive bladder cancer [65]. The *HMMR*, which was as the downstream gene upregulated by testis-specific protein Y-encoded demonstrated that it could be involved in the initiation and development of hepatocellular carcinoma via the activation of *HA-HMMR* signaling cascade [66].

Our results had shown that the expression of *HMMR* was higher in hepatocellular carcinoma tissues than normal liver tissues on mRNA levels and protein levels, and high expression of *HMMR* in liver hepatocellular carcinoma patients was an adverse prognostic factor. The genetic alteration of *HMMR* in liver cancer such as arm-level gain and high amplification could be found in our results, and further analysis indicated that high expression of *HMMR* could predict the elevated infiltration levels of B cells, CD8+ T cells, CD4+ T cells, macrophages, neutrophils, and dendritic cells. We inferred that *HMMR* could affect the activation and polarization of macrophages, especially for the M2 subtype. M2 macrophages were regarded as “renegade” immune cells which contributed to poor prognosis in liver hepatocellular carcinoma and promote cancer invasiveness [67]. In our study, *HMMR* was found to be positively correlated with M2 gene markers (CD163, VSIG4, and MS4A4A). These results suggested that *HMMR* might induce macrophage-related immune response by activating M2 subsets. The mechanism of *HMMR* in liver cancer is worthy of further testing.

The pituitary tumor transforming gene-1 (*PTTG1*) is a ubiquitously expressed regulator of sister-chromatid separation, and it also acts as the transcription factor [68]. In different types of cancer, including gastrointestinal tumors, urological tumors, and gynecologic tumors, the upregulation of *PTTG1* was related to unfavorable tumor phenotype and adverse prognosis [69–73]. The prior study had shown that the expression of the *PTTG1* in HepG2 and SMMC-7721 cells were higher than L02 cells. SiRNA knockdown of *PTTG1* induced the transformation in expression of P21 and P53 in HepG2 and SMMC-7721 cells [74]. Interestingly, in our study, the *PTTG1* was not enriched in the pathway of P53. The mechanism of *PTTG1* in liver cancer is worthy of an in-depth study.

Fujii et al. [75] reported that the PTTG1 was obviously overexpressed in hepatocellular carcinoma, which was consistent with our results that the mRNA expression levels and protein levels of *PTTG1* were higher in liver cancer tissues than normal liver tissues. The high expression of *PTTG1* was an adverse factor in survival rates of liver hepatocellular carcinoma patients. *PTTG1* SCNA was closely relevant to immune cell infiltration level, including arm-level gain and high amplification. *PTTG1* expression was positively associated with immune cells. The prior research identified that *PTTG1* was upregulated in T cell proliferation [76], and this was consistent with our results. We hypothesized that *PTTG1* was closely related to the immune response, and the mechanism of *PTTG1* in liver cancer is needed to be further explored.

The threonine tyrosine kinase (*TTK*) gene is located on chromosome 6q13-q21 and encodes a serine/threonine and tyrosine protein kinase. The *TTK* is an important component of the spindle of assembly checkpoint that ensures the fidelity of chromosome segregation [77]. The previous study had shown that elevated of *TTK* could cause centrosome enlargement and chromosomal instability, leading to tumorigenesis [78]. The *TTK* could be hardly detected in normal tissues, via Northern blot, except the testis and placenta [79]. However, high expression levels of *TTK* could be detected in different types of cancer, including glioblastoma, esophageal cancer, and breast cancer [80–82]. The prostate cancer patients with high expression levels of *TTK* had a shorter time to relapse [83]. The prior research suggested the *TTK* could regulate the TGF-β signaling pathway [84]. The new research had speculated that *TTK* could regulate the proliferation and apoptosis of cancer cells via Akt-mTOR signaling pathway [85]. Liu et al [86] reported that *TTK* was overexpressed in 77.63% (118/152) hepatocellular carcinoma specimens.

In our study, the *TTK* was overexpressed in liver hepatocellular carcinoma tissues on mRNA and protein levels; liver hepatocellular carcinoma patients with high expression levels of *TTK* had lower survival rates. The *TTK* SCNA was focused on deep deletion. *TTK* expression was positively correlated with the infiltration levels of B cells, CD8+ T cells, CD4+ T cells, macrophages, neutrophils, and dendritic cells. A prior study indicated that *TTK* mutations presented the strongest association with elevated PD-L1 expression [87]. Interestingly, in our study, the *TTK* did not present a strong association with the PD-L1. Nonetheless, understanding the exact role of *TTK* in PD-L1 regulation is required to further test.

The fostamatinib was the spleen tyrosine kinase (SYK) inhibitor, and it was able to inhibit both parental and sorafenib-resistant (SR) HCC cell lines in vitro and xenograft models [88]. In our study, the fostamatinib interacted with the *CDK1* and *TTK*, suggesting that *CDK1* and *TTK* might be potential drug targets for fostamatinib in anti-HCC therapy. The new study suggested that the inhibition of cyclin E1 by the cyclin-dependent kinase inhibitors dinaciclib and alvocidib (flavopiridol) could suppress HCC cell growth by inducing apoptosis and enhance the killing function of regorafenib and sorafenib in vitro and vivo [89]. In our results, the dinaciclib and alvocidib interacted with the *CDK1*. The rucaparib (AG014699), which was the inhibitor of the poly (ADP-ribose) polymerase-1 (PARP-1), might induce the apoptosis of HepG2 cells through the mitochondrial pathway and induced the migration of HepG2 cells by upregulating the PTEN and increasing the TIMP-3/MMP-3 ratio [90]. The traditional chemotherapeutic drugs (epirubicin, cyclophosphmide, and fluorouracil) played the important roles in treatment of liver cancer [91]. In our study, the *HMMR* had relationship with the epirubicin, cyclophosphmide, and fluorouracil. The pharmacological mechanisms between the hub genes and drugs are needed to be further explored.

The main restriction of our study was only at the level of bioinformatics analysis. So it was in urgent need of cytological experiments, animal experiments, and drug trials, etc., to identify these hub genes in liver cancer.

## Conclusions

To conclude, 168 DEGs was identified in liver cancer by integrated analysis in our study, which contained 41 hub genes. Four of these hub genes, including *CDK1*, *HMMR*, *PTTG1*, and *TTK*, were filtered out as potential biomarkers for diagnosis and prognosis of liver cancer. The expressions of *CDK1*, *HMMR*, *PTTG1*, and *TTK* were closely related to the immune cell infiltration and signaling pathway activation. Meanwhile, the *CDK1*, *HMMR*, and *TTK* had close interaction with new types of anticancer agents and traditional chemotherapy drugs. Therefore, laboratory and clinical research are needed to identify our results associated with pathogenesis of liver cancer, which can offer the last and accurate information for the prevention and therapy of liver cancer.

## Data Availability

The datasets used and analyzed in the study are available from the corresponding author on reasonable request. The datasets analyzed in the study could be found in the GEO portal (https://www.ncbi.nlm.gov/geo) and TCGA portal (https://portal.gdc.cancer.gov/).
